# Retinal nerve fiber hypertrophy in ataxia of Charlevoix-Saguenay patients

**Published:** 2011-07-13

**Authors:** Luis E Pablo, Elena Garcia-Martin, Jose Gazulla, Jose M Larrosa, Antonio Ferreras, Filippo M Santorelli, Isabel Benavente, Ana Vela, Miguel A Marin

**Affiliations:** 1 Ophthalmology Department, Miguel Servet University Hospital, Zaragoza, Spain; 2 Aragones Institute of Health Sciences, Zaragoza, Spain; 3 Neurology Department, Miguel Servet University Hospital, Zaragoza, Spain; 4 Neurogenetics Laboratory, Istituto di Neuropsichiatria Infantile, IRCCS Fondazione Stella Maris, Calambrone, Italy; 5 Service of Clinical Neurophysiology, Hospital San Jorge, Huesca, Spain; 6 Service of Radiology, Hospital Universitario Miguel Servet, Zaragoza, Spain

## Abstract

**Purpose:**

To present full ophthalmologic examination and retinal nerve fiber layer (RNFL) photographs of autosomal recessive spastic ataxia of Charlevoix-Saguenay (ARSACS) patients showing significant increases in RNFL thickness compared to healthy subjects, but without myelinated retinal fibers.

**Methods:**

The study design was observational case series. Ten eyes of five patients with molecular confirmation of ARSACS underwent a full ophthalmologic examination that included clinical history, visual acuity, biomicroscopy of the anterior segment, gonioscopy, Goldmann applanation tonometry, central corneal ultrasonic pachymetry, ophthalmoscopy of the posterior segment, standard automatic perimetry (Humphrey field), simultaneous stereophotographs of the optic disc after mydriasis, a series of five red-free digital fundus photographs for RNFL evaluation, topographic analysis of the optic disc using the Heidelberg retina tomography, and measurement of peripapillary RNFL thickness with Cirrus optical coherence tomography.

**Results:**

All patients showed abnormal visual fields, normal optic discs with a mild to strikingly increased visibility of RNFL in color stereophotographs, normal Heidelberg tomography, and moderate to markedly increased RNFL thickness in Cirrus tomography (average thickness ranging from 119 μm to 220 μm).

**Conclusions:**

We found evidence of RNFL hypertrophy in ARSACS patients that may have been interpreted as hypermyelinated retinal fibers in previous reports. A revision of ARSACS diagnostic criteria, particularly with regard to retinal alterations, is necessary.

## Introduction

Autosomal recessive spastic ataxia of Charlevoix-Saguenay (ARSACS) is a highly prevalent neurodegenerative disease in the Charlevoix-Saguenay-Lac-Saint-Jean region of the Province of Quebec (carrier frequency 1/22) [1]. In people from the region or with recent ancestors from there, the disease is typically characterized by early-onset (age 12–18 months) difficulty in walking and unsteadiness in gait. In individuals with ARSACS inherited through distant ancestors, onset is often delayed until later childhood and even adulthood. Ataxia, dysarthria, spasticity with extensor plantar reflexes, distal muscle wasting, sensorimotor neuropathy, and horizontal gaze-evoked nystagmus are the most frequent progressive neurologic signs [2,3]. Neuroimaging reveals atrophy of the superior vermis, cervical spinal cord, cerebello, and cerebral cortex [4].

ARSACS subjects from the Province of Quebec become wheelchair-bound at an average age of 41 years; cognitive skills are preserved in the long term, because individuals remain able to perform daily living tasks late into adulthood. Death commonly occurs in the sixth decade. Spastic Ataxia Charlevoix-Saguenay (*SACS*) is the gene most frequently associated with ARSACS (cytogenetic location: 13q12; molecular location on chromosome 13: base pairs 23,902,964 to 24,007,840). Canadian patients are usually homozygotes or compound heterozygotes for two founder mutations [5,6]. Although initial descriptions of the disease were confined to Quebec, genetically confirmed ARSACS has now been reported in individuals from France, Tunisia, Italy, Spain, Japan, and Turkey. Its true worldwide incidence remains unknown, because underdiagnosis is likely [5].

Concerning ARSACS, several authors have described yellow streaks of hypermyelinated fibers that focally cover the retinal vessels, emanating radially from the edges of the optic fundus and extending into the peripheral retina, although these streaks are uncommon in individuals with ARSACS who have European or Turkish heritage. Japanese individuals have been described with cognitive impairment, but without spasticity or retinal streaks [7]. Nevertheless, we present here the cases of five patients (ten eyes) with full ophthalmologic examination and retinal nerve-fiber layer (RNFL) photographs showing significant increases in RNFL thickness compared to healthy subjects and RNFL hypertrophy corresponding to the radial yellow peripapillary streaks that have been observed clinically.

## Methods

Five unrelated patients with genetically confirmed ARSACS are presented. All of them showed spasticity in the inferior limbs, ataxia, abnormal reflexes, pes cavus, and hammer toe. Patient characteristics are shown in Table 1. Molecular study demonstrated a compound heterozygous mutation in the giant exon of the *SACS* gene (13q12) in all patients, except in patient 4, who presented a homozygous mutation.

**Table 1:** Epidemiological characteristics and qualitative aspects of optic disc and retinal nerve fiver layer using stereophotographs, OCT and HRT, and visual field test results, for the five patients studied.

**Patient**	**Sex**	**Age**	**Stereophotographs**	**RNFL**	**OCT**	**HRT**	**Visual field**
1	F	48	Normal optic disc.	Moderately increased visibility	RNFL Thickness above normal values	Normal	Abnormal (general reduction of sensitivity).
			Moderately increased visibility of RNFL peripapillary.	PSD: 4.43 RE; 5.36 LE.			
2	M	38	Normal optic disc.	Moderately increased visibility	RNFL Thickness above normal values	Normal	Mild non-specific defects (localized defects).
			Mildly increased visibility of RNFL peripapillary.	PSD: 5.02 RE; 2.11 LE.			
3	F	40	Normal optic disc.	Strikingly increased visibility	RNFL Thickness above normal values	Normal	Abnormal (general reduction of sensitivity).
			Moderately increased visibility of RNFL peripapillary.	PSD: 4.12 RE; 6.17 LE.			
4	F	41	Normal optic disc.	Moderately increased visibility	RNFL Thickness above normal values	Normal	Mild non-specific defects (localized defects).
			Moderately increased visibility of RNFL peripapillary.	PSD: 2.27 RE; 5.79 LE.			
5	F	57	Normal optic disc.	Strikingly increased visibility	RNFL Thickness above normal values	Normal	Abnormal (general reduction of sensitivity).
			Moderately increased visibility of RNFL peripapillary.	PSD: 7.68 RE; 5.58 LE.			

Abbreviations: F, female; M, male; RNFL, retinal nerve fiver layer; OCT, Optical coherence tomography; HRT, Heidelberg Retina Tomograph; PSD, pattern standard deviation; RE, right eye; LE, left eye.

The ten eyes of five subjects underwent a full ophthalmologic examination that included visual acuity, biomicroscopy of the anterior segment using a slit-lamp, gonioscopy, Goldmann applanation tonometry, central corneal ultrasonic pachymetry (model DGH 500; DGH Technology, Exton, PA), and ophthalmoscopy of the posterior segment.

Standard automatic perimetry was performed in the five patients using a Humphrey field analyzer model 750i (Carl Zeiss Meditec, Dublin, CA) with the SITA Standard 24–2 program. Near addition using autorefractor Topcon RM-8800 (Topcon Medical Systems, Paramus, NJ) and optical correction of presbyopia), was added to the subject’s refractive correction.

Simultaneous stereophotographs of the optic disc were taken after mydriasis (0.5% tropicamide; Alcon Laboratories Inc., Fort Worth, TX) using a Canon CF-60UV fundus camera (Canon Inc., Tokyo, Japan).

A series of five red-free digital fundus photographs (Canon CF-60UVi, with a Canon EOS D60 digital camera and a specific filter with maximum transmission of light at 490 nm; Canon Inc., Tokyo, Japan) of each eye was acquired for RNFL evaluation. One RNFL monochromatic photograph was centered on the optic disc and two others on each arcuate zone. The digital image processor stored the digital presentation of the red-free fundus photographs with a resolution of 3,072×2,048 pixels for 60°.

Topographic analysis of the optic disc was performed using the Heidelberg Retina Tomograph (HRT) 3 (Heidelberg Engineering, Dossenheim, Germany), which provides topographic measurements of the optic nerve head derived from 16 to 64 optical sections to a depth of 4 mm, depending on the longitudinal field of view. The spherical equivalent refractive error of each eye was adjusted in the dioptric ring of the HRT. To correct magnification errors, keratometric readings were entered into the software. Topographic images were analyzed using the Advanced Glaucoma Analysis 3.0 software (Heidelberg Engineering, Dossenheim, Germany). To be accepted, all scans had to have an interscan standard deviation of less than 30 µm. The margin of the optic discs was manually traced by the same ophthalmologist. Global stereometric parameters were investigated in each patient.

The Zeiss Cirrus Optical Coherence Tomography (OCT; software version: 4.0.1.3) was used to measure peripapillary RNFL thickness. The optic disc protocol generates 200×200 cube images measuring a 6 mm×6 mm area around the optic nerve. In each scan, average RNFL thickness, quadrant RNFL thickness (superior, inferior, temporal, and nasal), and 12 clock hours of 30° RNFL thickness were analyzed and compared with the normative database of this instrument.

All procedures were evaluated by a neuro-ophthalmology specialist.

## Results

**Figure 1:**
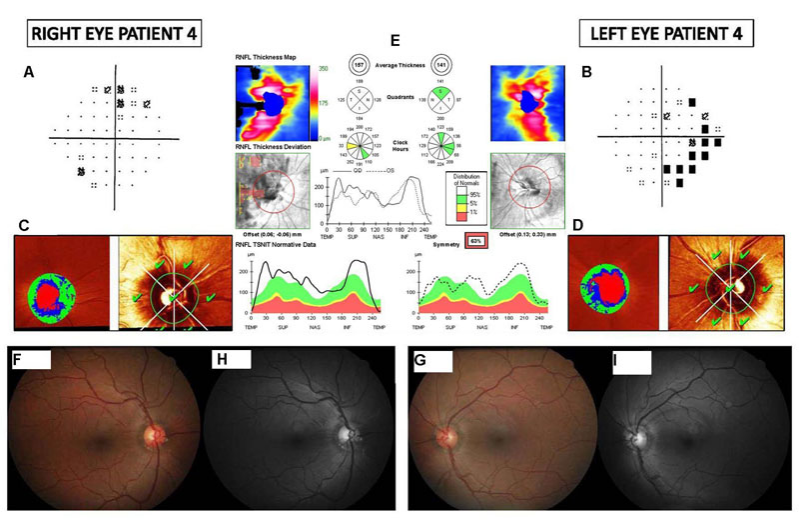
Figure 1. Patient 4 ophthalmologic exploration. Representation of visual field of right and left eye (**A** and **B**, respectively), Heidelberg retinal (**C** and **D**, respectively) and optical coherence tomographic assessments for both eyes (**E**). The images **F** and **G** represent optic disc color stereophotographs of right and left eye, respectively. The images **H** and **I** show retinal nerve fiber layer monochromatic photographs for both eyes. Results reflect mild nonspecific defects in the visual field, normal optic nerve morphology, and an increase in global retinal nerve fiber layer thickness and density.

**Figure 2:**
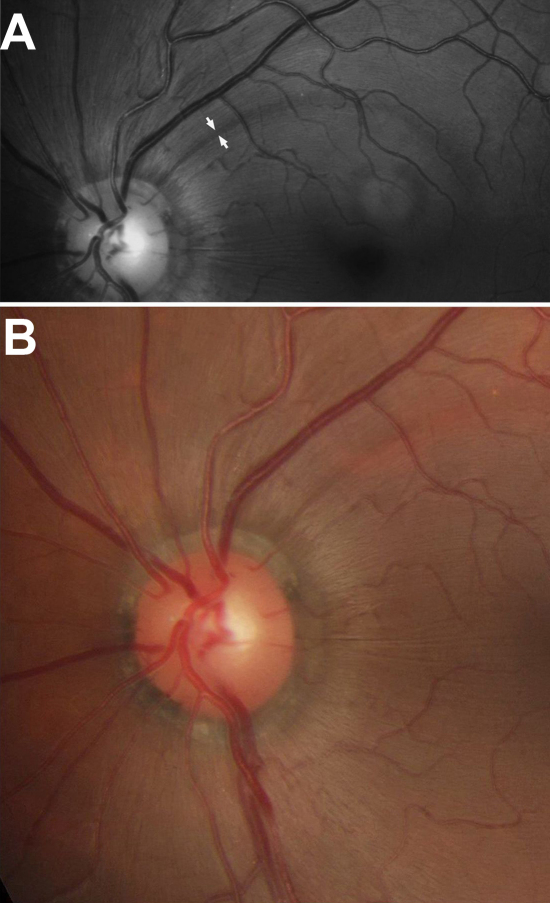
Monochromatic and stereophotographs in autosomal recessive spastic ataxia of Charlevoix-Saguenay 198 patient. **A**: A monochromatic photograph of retinal nerve fiber layer in patient 4 (left eye) shows increased visibility of fibers and a thin sector defect (included between arrows). **B**: A stereophotograph of the same eye shows the telltale yellow discoloration of the retinal nerve fiber layer streaks.

**Figure 3:**
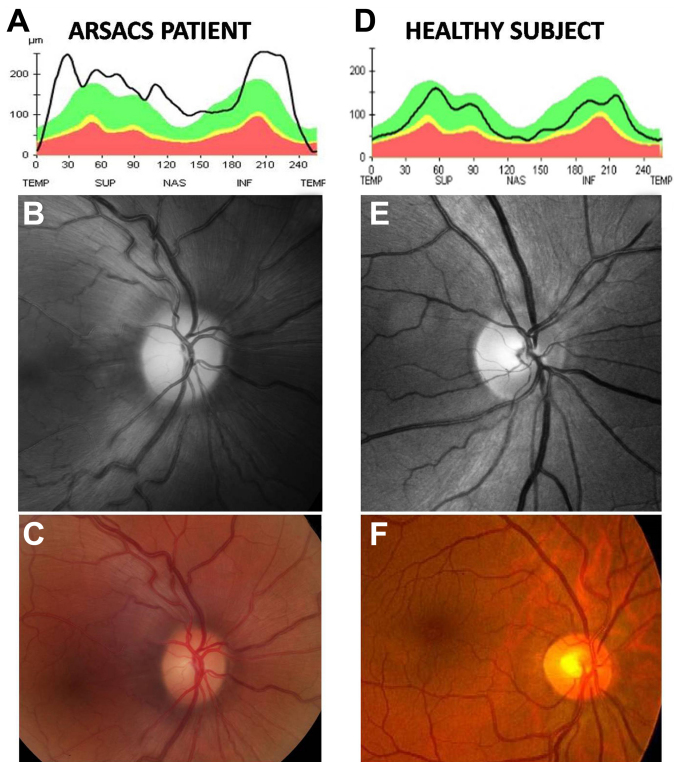
Comparison of an autosomal recessive spastic ataxia of Charlevoix-Saguenay patient and a healthy subject. The patient (left) shows an increase of retinal nerve fiber layer thickness in the optical coherence tomograph (**A**), and moderately increased visibility of the retinal nerve fiber layer in the stereophotograph (**B**) of the optic disc and in the monochromatic red-free digital fundus photograph (**C**), compared with healthy eye (**D**-**F**).

All patients showed abnormal visual fields, with mild to severe nonspecific localized defects in two patients and general reduction of sensitivity in three patients, a mean deviation (MD) index between −3.24 and −21.99, and a pattern standard deviation (PSD) between 2.27 and 7.68 (p<0.05 in all eyes). Patient 1 showed low reliability, probably due to nystagmus and low visual acuity. Optic disc color stereophotographs displayed normal optic discs with mild to strikingly increased visibility of RNFL (Table 1, Figure 1). RNFL monochromatic photographs showed moderate or strikingly increased visibility (Table 1), but the left eye of patient 4 presented a thin sector defect (Figure 2). RNFL evaluation using OCT presented moderate to markedly increased thicknesses (Figure 3), ranging from 119 to 220 microns in average thickness (Table 2), which was well above normal values (96.0±7.7 microns) [7]. Examination with a HRT III topographer by sectors was within normal limits in all cases (Table 1); however, all explorations showed mean RNFL thicknesses under 0.20 mm (Table 2). The reason underlying this was the method of measurement-to a depth of 4 mm. An oversized RNFL, evident in all the patients, probably resulted in the miscalculation of RNFL thickness measurement.

**Table 2:** Mean average of both eyes in patients for visual field, OCT and HRT measurements.

		**Patient 1**	**Patient 2**	**Patient 3**	**Patient 4**	**Patient 5**
VISUAL FIELD	MD	−9.45	−4.97	−8.24	−6.41	−19.81
	MSD	4.89	3.56	5.14	4.03	7.13
OCT PARAMETERS (µm)	RNFL Average thickness	149	207	141	125.5	124
	RNFL Superior thickness	165	189.5	158	134.5	151.5
	RNFL Nasal thickness	133	134.5	109.5	84	90.5
	RNFL Inferior thickness	192	250	181.5	172	166.5
	RNFL Temporal thickness	106	256	115	112	89
HRT PARAMETERS	Rim Area	2.14	2.58	1.98	1.92	1.99
	Rim volume	0.31	0.55	0.25	0.46	0.35
	Linear Cup/Disc Ratio	0.43	0.39	0.55	0.56	0.5
	Cup Shape Measure	−0.25	−0.24	−0.19	−0.24	−0.23
	Height Variation Contour	0.38	0.33	0.32	0.44	0.38
	Mean RNFL thickness	0.1	0.2	0.16	0.2	0.18

Abbreviations: MD, mean deviation; MSD, mean standard deviation; HRT, Heidelberg Retina Tomograph; OCT, Optical coherence tomography; RNFL, retinal nerve fiver layer.

## Discussion

Previous authors have reported retinal hypermyelinated fibers to be a minor diagnostic criterion for ARSACS, but we found evidence of RNFL hypertrophy in these patients that may have been interpreted as hypermyelinated retinal fibers in those previous reports. As such, we suggest a revision of ARSACS diagnostic criteria, particularly with regard to retinal alterations.

We found that RNFL increased, but not the myelinated fibers radiating from the optic disk. These findings suggest the need to check the supposed hypermyelinated retinal fibers to determine criteria to establish ARSACS diagnosis. In our opinion, some of the fundus images described as hypermyelinated retinal fibers in the bibliography may be a RNFL increase as our patients showed [4], so we recommend that experts review these published photographs and perform a complete neuro-ophthalmologic examination using stereophotographs, RNFL photographs, and analysis with digital image analysis devices in ARSACS patients.

HRT measurements showed mean RNFL thicknesses under 0.20 mm. We think that RNFL hypertrophy may be the reason of these findings in HRT measurement, so the normative database for HRT does not apply to RNFL measurement with this device in ARSACS patients.

Ultrastructural observations have not corroborated the hypothesis that hypermyelinated fibers constitute the basic pathophysiology of this lesion in ARSACS. In addition, nerve biopsies in patients published to date have only revealed a depletion of myelinated fibers, but not hypermyelinated fibers [8]. A hypothesized role for the SACS gene is nerve fiber development, as MRI scans in ARSACS patients suggest [4], causing RNFL hypertrophy and alterations in the central nervous system.

Our results suggest retinal streaks caused by increases in RNFL density, but diagnostic histology of pathology specimens from deceased patients would strengthen this argument.

## References

[1] Engert JC, Bérubé P, Mercier J, Doré C, Lepage P, Ge B, Bouchard JP, Mathieu J, Melançon SB, Schalling M, Lander ES, Morgan K, Hudson TJ, Richter A (2000). ARSACS, a spastic ataxia common in northeastern Québec, is caused by mutations in a new gene encoding an 11.5-kb ORF.. Nat Genet.

[2] De Braekeleer M, Giasson F, Mathieu J, Roy M, Bouchard JP, Morgan K (1993). Genetic epidemiology of autosomal recessive spastic ataxia of Charlevoix-Saguenay in northeastern Quebec.. Genet Epidemiol.

[3] Dupré N, Bouchard JP, Brais B, Rouleau GA (2006). Hereditary ataxia, spastic paraparesis and neuropathy in the French-Canadian population.. Can J Neurol Sci.

[4] Gerwig M, Krüger S, Kreuz FR, Kreis S, Gizewski ER, Timmann D (2010). ARSACS outside Quebec Characteristic MRI and funduscopic findings help diagnose.. Neurology.

[5] El Euch-Fayache G, Lalani I, Amouri R, Turki I, Ouahchi K, Hung WY, Belal S, Siddique T, Hentati F (2003). Phenotypic features and genetic findings in sacsin-related autosomal recessive ataxia in Tunisia.. Arch Neurol.

[6] Ogawa T, Takiyama Y, Sakoe K, Mori K, Namekawa M, Shimazaki H, Nakano I, Nishizawa M (2004). Identification of a SACS gene missense mutation in ARSACS.. Neurology.

[7] Garcia-Martin E Pinilla I Idoipe M Fuertes I Pueyo V Intra and interoperator reproducibility of retinal nerve fibre and macular thickness measurements using Cirrus Fourier-domain OCT. Acta Ophthalmol 2010 21106044 10.1111/j.1755-3768.2010.02045.x

[8] Takiyama Y (2006). Autosomal recessive spastic ataxia of Charlevoix-Saguenay.. Neuropathology.

